# Lower plasma PCSK9 in normocholesterolemic subjects is associated with upregulated adipose tissue surface‐expression of LDLR and CD36 and NLRP3 inflammasome

**DOI:** 10.14814/phy2.14721

**Published:** 2021-02-01

**Authors:** Yannick Cyr, Valérie Lamantia, Simon Bissonnette, Melanie Burnette, Aurèle Besse‐Patin, Annie Demers, Martin Wabitsch, Michel Chrétien, Gaétan Mayer, Jennifer L. Estall, Maya Saleh, May Faraj

**Affiliations:** ^1^ Institut de recherches cliniques de Montréal (IRCM) Montréal QC Canada; ^2^ Faculty of Medicine Université de Montréal Montréal QC Canada; ^3^ Montreal Diabetes Research Center (MDRC) Montréal QC Canada; ^4^ Institut de cardiologie de Montréal (ICM) Montréal QC Canada; ^5^ Department of Pediatrics and Adolescent Medicine Ulm University Hospital Ulm Germany; ^6^ Ottawa Health Research Institute (OHRI) Ottawa ON Canada; ^7^ Faculty of Pharmacy Université de Montréal Montréal QC Canada; ^8^ Department of Medicine McGill University Montréal QC Canada; ^9^ Department of Life Sciences and Health The University of Bordeaux Bordeaux France

**Keywords:** adipose tissue and systemic inflammation, apoB‐lipoproteins, cardiometabolic risk, plasma apoB‐to‐PCSK9

## Abstract

**Background:**

LDL‐cholesterol lowering variants that upregulate receptor uptake of LDL, such as in *PCSK9* and *HMGCR*, are associated with diabetes via unclear mechanisms. Activation of the NLRP3 inflammasome/interleukin‐1 beta (IL‐1β) pathway promotes white adipose tissue (WAT) dysfunction and type 2 diabetes (T2D) and is regulated by LDL receptors (LDLR and CD36). We hypothesized that: (a) normocholesterolemic subjects with lower plasma PCSK9, identifying those with higher WAT surface‐expression of LDLR and CD36, have higher activation of WAT NLRP3 inflammasome and T2D risk factors, and; (b) LDL upregulate adipocyte NLRP3 inflammasome and inhibit adipocyte function.

**Methodology:**

Post hoc analysis was conducted in 27 overweight/ obese subjects with normal plasma LDL‐C and measures of disposition index (DI during Botnia clamps) and postprandial fat metabolism. WAT was assessed for surface‐expression of LDLR and CD36 (immunohistochemistry), protein expression (immunoblot), IL‐1β secretion (AlphaLISA), and function (^3^H‐triolein storage).

**Results:**

Compared to subjects with higher than median plasma PCSK9, subjects with lower PCSK9 had higher WAT surface‐expression of LDLR (+81%) and CD36 (+36%), WAT IL‐1β secretion (+284%), plasma IL‐1 receptor‐antagonist (+85%), and postprandial hypertriglyceridemia, and lower WAT pro‐IL‐1β protein (−66%), WAT function (−62%), and DI (−28%), without group‐differences in body composition, energy intake or expenditure. Adjusting for WAT LDLR or CD36 eliminated group‐differences in WAT function, DI, and postprandial hypertriglyceridemia. Native LDL inhibited Simpson‐Golabi Behmel‐syndrome (SGBS) adipocyte differentiation and function and increased inflammation.

**Conclusion:**

Normocholesterolemic subjects with lower plasma PCSK9 and higher WAT surface‐expression of LDLR and CD36 have higher WAT NLRP3 inflammasome activation and T2D risk factors. This may be due to LDL‐induced inhibition of adipocyte function.

## 1 INTRODUCTION

Type 2 diabetes (T2D) and cardiovascular disease share many risk factors such as unhealthy lifestyle and obesity. Yet, while lowering plasma low‐density lipoprotein cholesterol (LDL‐C) is cardio‐protective, evidence over the past decade has recognized a role for common LDL‐C‐lowering variants, including loss‐of‐function variants in *PCSK9* (Proprotein Convertase Subtilisin/ kexin Type 9) and *HMGCR* (3‐hydroxy‐3‐methylglutaryl‐CoA reductase), in higher risk for diabetes (Ference et al., 2016; Lotta et al., 2016; Schmidt et al., 2016; Swerdlow et al., 2015). Similarly, long‐term use of statins that inhibit HMGCR is associated with a higher incidence of T2D (Corrao et al., 2014; Ridker et al., 2012; Sattar et al., 2010; Swerdlow et al., 2015). As these inherited or induced conditions lower plasma LDL‐C by increasing tissue‐uptake of LDL, a role for the LDL receptor (LDLR) pathway was proposed (Ference et al., 2016; Preiss & Sattar, 2015; Schmidt et al., 2016). However, mechanisms underlying higher risk for T2D with the upregulation of the LDLR pathway remain unclear.

White adipose tissue (WAT) dysfunction is believed to play a central role in the pathophysiology of T2D as it promotes elevated plasma lipids, particularly in the postprandial state, and their influx into peripheral tissues favoring systemic lipotoxicity, insulin resistance, and hyperinsulinemia. This in time favors β‐cell exhaustion, reduced insulin secretion, and hyperglycemia (Faraj, 2020; Gastaldelli et al., 2017). A lower insulin sensitivity combined with insufficient insulin secretion, measured as a lower disposition index (DI), is an independent predictor of the conversion of prediabetes to T2D across many ethnic groups and races (Lorenzo et al., 2010).

Over the past 10 years, we established a role for native LDL in the dysfunction of murine adipocytes and human subcutaneous WAT (Bissonnette et al., 2013; Lamantia et al., 2017). We also reported that elevated plasma numbers of apoB‐lipoproteins (i.e., plasma apoB) are associated with risk factors for T2D in overweight and obese subjects (Bissonnette et al., 2013, 2015; Bissonnette, Saint‐Pierre, et al., 2018; Lamantia et al., 2017; Wassef et al., 2015). However, the association of higher plasma apoB with WAT dysfunction and insulin resistance was strengthened by lower plasma PCSK9 (Wassef et al., 2015), suggesting a role for PCSK9‐regulated receptors, such as LDLR and CD36 (Demers et al., 2015; Schmidt et al., 2008) in metabolic dysfunction. More recently, we reported that higher fasting WAT‐surface expression of LDLR and CD36 in subjects with overweight and obesity was positively associated with insulin resistance and plasma interleukin 1 receptor antagonist (IL‐1Ra) (Cyr et al., 2020), which is a measure of the systemic activation of the interleukin 1 (IL‐1) signaling (Bissonnette et al., 2015).

A key sensor of metabolic stress in WAT that is implicated in the etiology of WAT dysfunction and T2D is the Nucleotide‐binding domain and Leucine‐rich repeat Receptor, containing a Pyrin domain 3 (NLRP3) inflammasome (Koenen et al., 2011; Skeldon et al., 2014; Stienstra et al., 2010; Vandanmagsar et al., 2011). Activation of the NLRP3 inflammasome leads to the secretion of IL‐1β (Swanson et al., 2019), which inhibits insulin signaling in adipocytes, pancreatic β‐cells, and hepatocytes (Skeldon et al., 2014; Stienstra et al., 2010; Vandanmagsar et al., 2011) and interferes with adipocyte differentiation (Stienstra et al., 2010). Activation of the inflammasome requires two signals, a *priming* signal that induces *NLRP3* and *pro*‐*IL*‐*1β* expression, followed by an *activation* signal that promotes the assembly of the inflammasome complex, activation of caspase‐1, cleavage of pro‐IL‐1β into IL‐1β (biologically active form) followed by the secretion of IL‐1β (Swanson et al., 2019). In macrophages, oxidized LDL (oxLDL) were reported to *prime* and *activate* the NLRP3 inflammasome in a mechanism dependent on CD36 (Sheedy et al., 2013), a scavenger receptor for native VLDL and LDL, oxLDL, and non‐esterified fatty acids (NEFAs) (Calvo et al., 1998; Glatz & Luiken, 2018)). More recently, both oxidized and native LDL were reported to upregulate the NLRP3 inflammasome in endothelial tubular cells, suggesting a role for LDLR (Rampanelli et al., 2017). Moreover, preliminary work from our lab demonstrates that native LDL induces IL‐1β secretion in human WAT (Bissonnette, Lamantia, et al., 2018). Taken together, this data suggests that the activation of the NLRP3 inflammasome may be a mechanism linking receptor‐mediated tissue‐uptake of LDL to metabolic risk in humans.

Accordingly, we ran post hoc analyses of two cohorts of overweight and obese subjects with normal plasma LDL‐C grouped based on median plasma PCSK9 per sex as PCSK9 regulates tissue‐surface expression of LDLR and CD36 (Demers et al., 2015; Schmidt et al., 2008). We tested the hypotheses that, compared to subjects with higher plasma PCSK9, subjects with lower plasma PCSK9, identifying those with higher WAT surface expression of LDLR and CD36, have (a) higher WAT and systemic activation of NLRP3 inflammasome, and (b) higher risk factors for T2D; namely WAT dysfunction, insulin resistance, hyperinsulinemia, lower DI, and postprandial hypertriglyceridemia. We also examined whether native LDL directly regulates the NLRP3 inflammasome and adipocyte physiology in a human adipocyte model.

## 2 RESEARCH DESIGN AND METHODS

### 2.1 Study population

This work represents post hoc analysis of the baseline data of two registered clinical trials conducted at IRCM between 2010 and 2020 (ISRCTN14476404 and NCT04496154). Both studies enrolled men and postmenopausal women between 45 and 74 years, who were non‐smokers, sedentary (<2 h of structured exercise/week), and with low alcohol consumption (≤2 servings/day). The exclusion criteria included having ≥20% Framingham Risk Score, prior history of chronic disease (including cardiovascular disease or event, inflammatory disease, and diabetes), and medication affecting metabolism (Bissonnette et al., 2015; Bissonnette, Saint‐Pierre, et al., 2018; Cyr et al., 2016; Lamantia et al., 2017, 2019; Wassef et al., 2015). All participants signed consent forms before initiating the two studies, which included the conservation and use of their biological samples for 10 years after the study end. The consent forms were approved by the ethics board at IRCM and respected the Declaration of Helsinki principles. Subject included in this analysis had a BMI of 25–44 kg/m^2^ and normal plasma LDL‐C (LDL‐C < 3.5 mmol or <75th percentile in the Canadian population (Connelly et al., 1999)) measured on two testing days (1–4 weeks apart). Included subjects had measures of insulin sensitivity and secretion and postprandial plasma clearance of fat and had preserved WAT samples and conditioned medium for the additional measurement of surface‐expression of LDLR and CD36 and WAT NLRP3 inflammasome activity (N = 27, 15 women and 12 men).

### 2.2 Anthropometrics and metabolic parameters

All subjects underwent a 4‐week weight stabilization period before initiating metabolic testing (±2 kg). Body composition was assessed by dual‐energy X‐ray absorptiometry (iDXA; GE Healthcare) and basal metabolic rate was measured by indirect calorimetry (Vmax Encore, CareFusion). Three‐day food records were completed by the subjects (two weekdays, one weekend) and verified by a dietitian for completion. Their nutritional analysis was conducted using The Food Processor software (version 11.3.285, ESHA Research). Plasma parameters were measured as follows: lipids and apoB by COBAS Integra 400 analyzer (Roche Diagnostic), apoB48 by ELISA (Fuji Films/WAKO), glucose by an automated analyzer (YSI Inc., InterScience), insulin and C‐peptide by radioimmunoassay kit (Millipore Corporation), PCSK9 by ELISA kit (Circulex), and IL‐1Ra by high‐sensitivity ELISA kit (R&D system, Minneapolis, MN, USA)(Bissonnette, Saint‐Pierre, et al., 2018; Bissonnette et al., 2013, 2015; Lamantia et al., 2017; Wassef et al., 2015).

### 2.3 Insulin sensitivity and secretion

On the first testing day, insulin sensitivity and secretion were measured by a gold‐standard technique using modified Botnia clamp as reported (Bissonnette et al., 2015; Bissonnette, Saint‐Pierre, et al., 2018; Cyr et al., 2020; Lamantia et al., 2017; Wassef et al., 2015). Briefly, subjects followed a 3‐day high carbohydrate diet to maximize glycogen storage, after which they underwent a 1‐h intravenous‐glucose tolerance test (IVGTT, 0.3 g glucose/kg) followed by a 3‐h hyperinsulinemic‐euglycemic clamp (75 mU insulin/m^2^/min). Insulin secretion was measured as glucose‐induced insulin and C‐peptide secretion during the 1st phase (first 10 min) and total of 60 min of the IVGTT. Insulin sensitivity was assessed during the steady state of the clamp (last 30 min) calculated as glucose infusion rate (GIR) divided by plasma insulin (termed M/I). The DI, which assesses insulin sensitivity and secretion concomitantly, was calculated as the 1^st^ phase or total glucose‐induced C‐peptide secretion multiplied by M/I (Lorenzo et al., 2010). Fasting index of insulin resistance was calculated as the Homeostatic Model Assessment of Insulin Resistance (HOMA‐IR) (Bissonnette et al., 2015; Bissonnette, Saint‐Pierre, et al., 2018; Cyr et al., 2020; Lamantia et al., 2017; Wassef et al., 2015).

### 2.4 Postprandial plasma clearance of fat and WAT biopsies

On the second testing day, 1–4 weeks after the first testing day, fasting WAT samples were collected from the hip by needle biopsy under local anesthesia (Xylocaine 20 mg/ml, AstraZeneca) and washed with cold antibiotic/antifungal‐supplemented HBSS buffer. Following that, subjects consumed a high‐fat meal composed of croissant, cheese, bacon, and brownies (600 kcal/m^2^, 68% fat, 36% saturated fat, 18% carbohydrate). Postprandial plasma clearance of fat was measured over 6 hours. In both clinical trials, when sufficient WAT samples were collected, one portion was immediately snap‐frozen in liquid nitrogen for mRNA and protein expression, a second portion was fixed overnight at 4°C in 4% paraformaldehyde, embedded in paraffin and cut into 5 µm sections, and a third portion was used fresh to measure WAT function ex vivo and collect WAT‐conditioned medium over 4 hours (Bissonnette et al., 2013; Cyr et al., 2016, 2020; Lamantia et al., 2017; Wassef et al., 2012).

### 2.5 WAT protein expression

WAT protein was collected and analyzed as previously reported (Cyr et al., 2020). Briefly, frozen WAT samples were crushed in liquid nitrogen, total proteins were extracted in radioimmunoprecipitation buffer (RIPA; Santa Cruz) and quantified by lipid‐insensitive CBQCA fluorescence‐based protein assay (Thermo Fisher). Protein samples were separated by SDS‐PAGE with equal loading verified by 1.0% TCE (Sigma T54801) stain‐free total protein visualization. Proteins were transferred onto 0.45 µm polyvinylidene difluoride membrane (PVDF; Millipore), blocked in 5% non‐fat dry milk in 0.1% Tween‐Tris buffer, and probed overnight with required antibodies as previously published (Cyr et al., 2020). Protein bands were revealed by chemiluminescence (SuperSignal West Femto Substrate, Thermo Fisher) in the ChemiDoc XRS+imaging system (Bio‐Rad). Band intensities were quantified using Image Lab software (Version 5.2.1; Bio‐Rad). Normalization of WAT protein expression was in accordance with published guidelines (Murphy & Lamb, 2013). Inter‐gel variability was mitigated by the use of an internal control made from pooled WAT from five subjects as described (Cyr et al., 2020). Samples were ran in duplicate on two gels and % CTL of the two gels was averaged and presented.

### 2.6 WAT surface‐expression of LDLR and CD36

**FIGURE 1:**
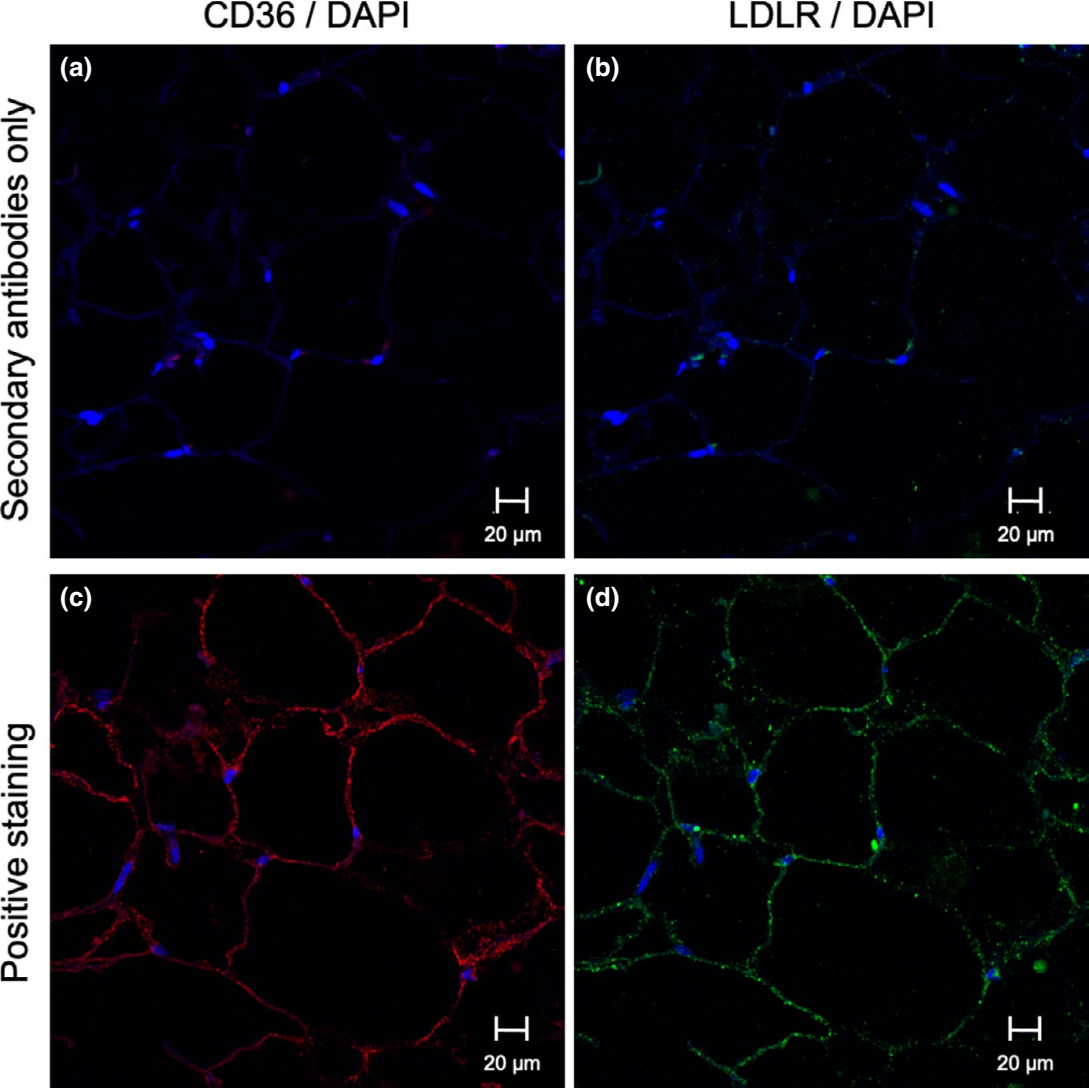
Representative plasma membrane localization of CD36 and LDLR by immunohistofluorescence staining in WAT. Specificity of LDLR and CD36 detection in WAT was verified by secondary antibody‐only staining for CD36 (Alexa Fluor 555 anti‐rabbit IgG) (a), and LDLR (Alexa Fluor 647 anti‐rabbit IgG) (b), used as negative control; Positive staining (primary and secondary antibodies) of WAT section for CD36 (c) and LDLR (d) All samples counterstained with nuclear stain DAPI. A‐B represent the same WAT section, C‐D represent the same WAT section, all from the same subject

This was measured using immunohistofluorescence (IHF) and confocal microscopy as previously reported (Cyr et al., 2020). Briefly, slide‐mounted WAT sections were deparaffinized and heat‐induced epitope retrieval (HIER) was performed (PT Module, Thermo Fisher), before blockage in BSA 0.1%. Sections were co‐incubated with goat anti‐human LDLR and rabbit anti‐human CD36 (Novus Biologicals, CO) in BSA 0.1% overnight at 4°C and then co‐incubated with secondary antibodies (Alexa Fluor 555 anti‐rabbit IgG, Alexa Fluor 647 anti‐goat IgG, Invitrogen) and counterstained with the nuclear dye DAPI (0.5 µg/ml) in BSA 0.1%. Slides were mounted in Vectashield antifade mounting medium (Vector Labs). Immunofluorescence acquisition was performed on a Zeiss LSM‐710 confocal microscope. Image analysis was performed using FIJI open‐access software (GitHub) and computed as corrected total fluorescence (CTF) of at least eight non‐overlapping fields per subject. A representative section of WAT with plasma membrane localization of CD36 and LDLR is presented in Figure 1. To reduce inter‐assay variability in immunofluorescence analyses, an internal control (CTL) made of consecutive sections of one subject was ran with each analysis and subject data were normalized to it and expressed as a percent of CTL.

### 2.7 WAT function and IL‐1β secretion

WAT function was measured ex vivo in fresh WAT samples as in situ lipoprotein lipase (LPL) activity. This technique assesses the overall hydrolysis of synthetic ^3^H‐triolein‐labeled‐triglyceride‐rich‐lipoproteins (^3^H‐TRL, 95% TGs, 1.27 mM TGs) and storage of generated ^3^H‐fatty acids as WAT ^3^H‐lipids over 4 hours. It is expressed as the mass of ^3^H‐TG hydrolyzed per milligram of WAT (Bissonnette et al., 2013, 2015; Cyr et al., 2016, 2020; Lamantia et al., 2017). WAT IL‐1β secretion was measured using AlphaLISA kits (Perkin Elmer) as the accumulation of IL‐1β in WAT‐conditioned medium over 4 hours (DMEM/F12 medium, Life Technologies) supplemented with 5% FBS (Wisent)). Each data point represents the mean of 2–4 WAT pieces (5–10 mg total) per well. WAT function represents the mean of 3–6 replicate wells and WAT IL‐1β secretion represents the mean of 2–3 replicate wells per subject.

### 2.8 Separation of subjects into groups with lower and higher plasma PCSK9

Included subjects were divided based on median plasma PCSK9 per sex to account for sex‐differences in measured parameters. This generated two groups with lower plasma PCSK9 (80.0–259.5 pg/ml in women and 155.2–211.1 pg/ml in men) and higher plasma PCSK9 (272.5–559.7 pg/ml in women and 213.6–305.6 pg/ml in men).

### 2.9 Effect of native human LDL on SGBS preadipocyte differentiation

Native LDL was isolated from healthy volunteers based on the following inclusion criteria: non‐smoker, not taking any medication affecting lipid metabolism, not having dyslipidemia or chronic diseases. Blood (60 mL) was drawn after an 8 h fast and plasma LDL was isolated by sequential ultra‐centrifugation, sterilized by filtration, stored in sterile saline, and used within 4 weeks as published (Bissonnette et al., 2013; Lamantia et al., 2017). Participants signed a consent form that was approved by the ethics board at IRCM in accordance with the Declaration of Helsinki principles. All LDL preparations were used anonymously.

Simpson‐Golabi Behmel‐syndrome (SGBS) cells are not transformed nor immortalized but have a high proliferation and differentiation capacity and are classically used as a human model of preadipocytes (Fischer‐Posovszky et al., 2008). Simpson‐Golabi Behmel‐syndrome cells are used as the yield of preadipocytes from subject WAT biopsies is too small for differentiation and experimentation. cells were maintained in 10% active FBS DMEM/F12 (Life Technologies) supplemented in pantothenate (2.17 µM), biotin (0.33 µM), 1% penicillin‐streptomycin, and gentamicin (0.25 µg/ml) (Fischer‐Posovszky et al., 2008). They were passaged at 80% confluence and plated (8000 cells/cm^2^) in 96‐well poly‐D‐lysine coated optical plates (Nunc). Differentiation was initiated 3‐days post‐plating (day 0, 90% confluency) and induced in albumin‐free medium by withdrawing FBS from the culture medium and supplementing the medium with an adipogenic cocktail (Fischer‐Posovszky et al., 2008). Preadipocytes were differentiated in the presence of saline 1.006 g/L as a control or in the presence of LDL in saline (0.05 −1.00 g apoB /L). At 7‐days of differentiation, cell number was measured by Hoechst stain (Life Technologies) and lipid accumulation by Nile Red staining (AdipoRed; Lonza Enterprises) using a SpectraMax I3 fluorometer (Molecular Devices). Images were developed using laser‐scanning confocal microscopy (LSM710, Zeiss). Adipocyte function was measured as in situ LPL activity over 4 hours using 1.27 mM of TGs substrate and expressed as the mass of ^3^H‐TG hydrolyzed per cell (Bissonnette et al., 2013; Faraj et al., 2004).

RNA was isolated from SGBS preadipocytes (day 0) and from mature adipocytes (6–7 days of differentiation) using TRIzol reagent (Qiagen). RNA (1 µg) was treated with DNaseI, reverse‐transcribed using the High Capacity Reverse Transcription Kit (Applied Biosystems), and quantified by real‐time PCR using SYBR Green PCR master mix with the Viaa7 RT‐PCR system (Life Technologies). Gene expression was normalized to beta‐2‐microglobulin (*B2M*) mRNA and expressed relative to normally differentiated adipocytes (CTL) using the ∆∆Ct threshold cycle method of normalization. The expression of NLRP3 was assessed in SGBS cells with and without *priming* with lipopolysaccharides (LPS, 0.1 to 1 ng/ml for 4 hours), which is a *priming* control for the NLRP3 inflammasome in macrophages (Swanson et al., 2019). Identical primers were used in SGBS cells and in subject WAT (primer sequences are available upon request to the corresponding author**)**.

### 2.10 Statistical analyses

**FIGURE 2:**
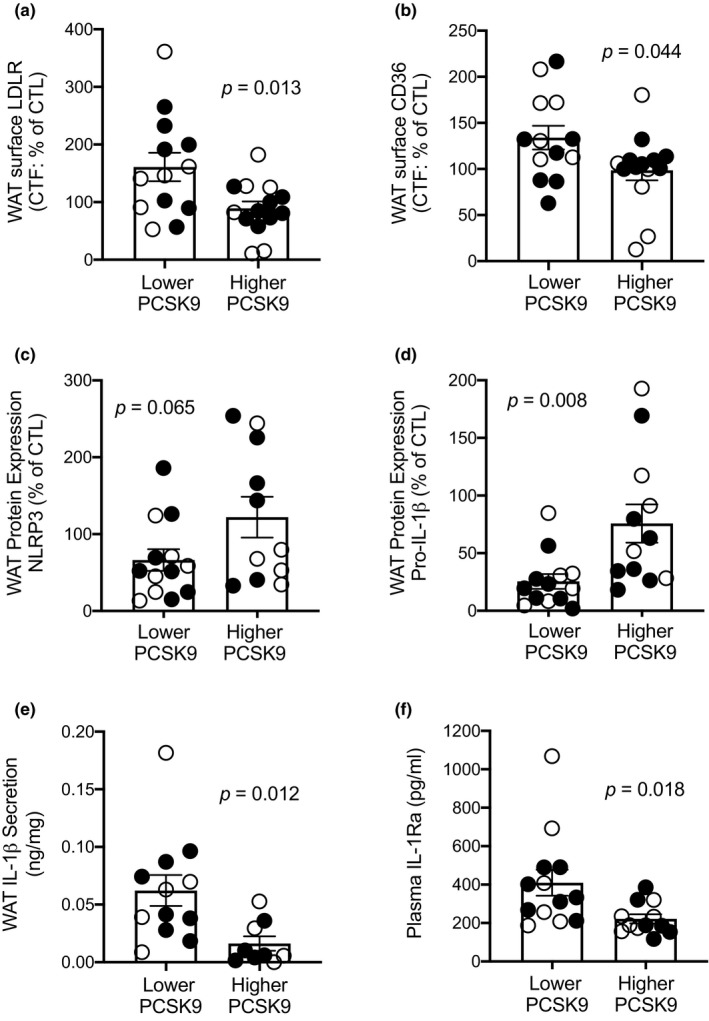
Subjects with lower PCSK9 have higher WAT surface LDLR and CD36 expression with upregulated NLRP3 inflammasome activity. Group difference in WAT surface LDLR (a) and CD36 (b), total WAT NLRP3 expression (c), pro‐IL‐1β expression (d), IL‐1β medium accumulation (e), and plasma IL‐1Ra (f). Data presented for N = 13 in the lower and N = 14 in the higher PCSK9 groups except for panel C where N = 11 in the higher PCSK9 group, panel D where N = 12 in the higher PCSK9 group, panel E where N = 12 in the lower and N = 9 in the higher PCSK9 group, and panel F where N = 12 in the higher PCSK9 group due to missing data. Women are presented as closed circles and men as open circles. Significant findings hold when performing non‐parametric sensitivity analysis

**FIGURE 3:**
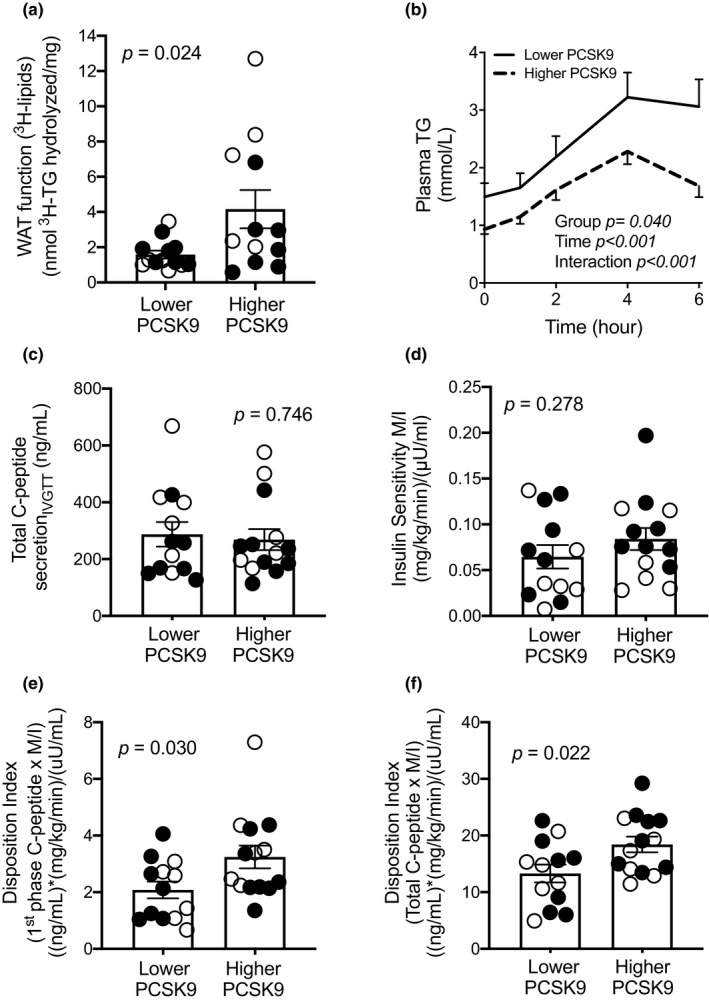
Subjects with lower PCSK9 have lower disposition index, impaired WAT function, and delayed postprandial TG clearance. Group differences in *ex vivo* WAT function (a), and postprandial plasma clearance of TGs (b), glucose‐induced total C‐peptide secretion (c), insulin sensitivity index M/I (d), first phase disposition index (E), and total disposition index (F). Data presented for N = 13 in the lower and N = 14 in the higher PCSK9 group in both groups except for panel A where N = 12 in the higher PCSK9 group due to insufficient WAT samples. Women are presented as closed circles and men as open circles. Significant findings hold when performing non‐parametric sensitivity analysis except for WAT function (*p* = 0.054)

**FIGURE 4:**
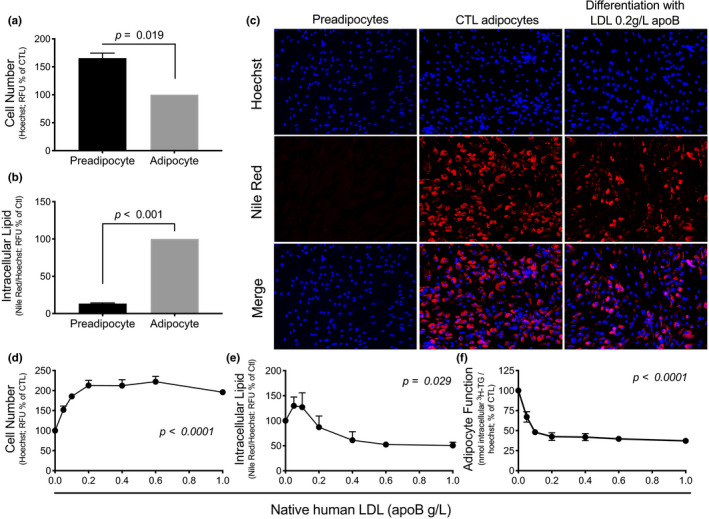
Native human LDL impairs differentiation and function of SGBS preadipocytes. The effect of differentiation of SGBS preadipocytes for 7 days on proliferation (a) and lipid‐droplet accumulation per cell (b). A representative experiment using laser‐scanning confocal microscopy (10×) to visualize cell number and lipid‐droplet accumulation in preadipocytes, 7d‐normally differentiated adipocytes (CTL), and 7d‐differentiated adipocytes in the presence of native human LDL (0.2 g apoB/L) (c). The effect of 7d‐differentiation of SGBS preadipocytes in the presence of LDL (0–1.0 g apoB/L) on cell number (d), lipid‐droplet accumulation per cell (e), and adipocyte function per cell (f). N = 3 different experiments measured in triplicates

**FIGURE 5:**
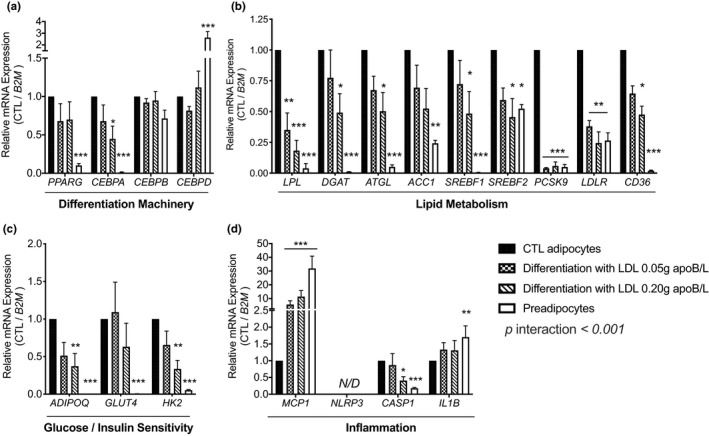
Native human LDL downregulate genes related to differentiation and function and upregulate *MCP*‐*1* expression during SGBS preadipocyte differentiation. Expression of genes related to differentiation (a), lipid metabolism (b), glucose uptake/utilization and insulin sensitivity (c), and inflammation (d) in SGBS preadipocytes and 7d‐differentiation of SGBS preadipocytes in the presence of native human LDL (0.05 or 0.20 g apoB/L) compared to normally differentiated adipocytes. mRNA are normalized to beta‐2‐microglobulin (*B2M*) and expressed relative to control adipocytes. Data were analyzed by 2‐way ANOVA with interaction and Dunnett correction for multiple comparisons. N = 4 experiments in duplicates. *p < 0.05, **p < 0.01, ***p < 0.001; compared to control adipocytes

Data are presented as the mean ± SEM. Student's *t*‐test was used for all group comparisons. Given the variability of some data, sensitivity analysis was performed using the non‐parametric Mann‐Whitney U test for intergroup differences in Table 1 and Figures 2 and 3. Univariate ANOVA was used to adjust group‐differences for covariates (LDLR, CD36, apoB, and NEFAs). One‐way ANOVA was used for univariate multiple analyses (Figure 4) and two‐way ANOVA with interaction for bivariate multiple analyses (group x time for plasma TG curves in Figure 3, gene x experimental condition in Figure 5). When interaction passed, unpaired *t*‐test was used to compare group differences or experimental conditions (N.B. *MCP*‐*1* expression was excluded from the interaction analysis as its high expression skewed the interaction analysis of the 20 genes in Figure 5). Dunnett correction was used to adjust for multiple comparisons in the ANOVA models. Statistical analyses were performed using SPSS V24 (IBM) and GraphPad Prism V8 (GraphPad Software). Significance was set at *p* < 0.05.

**TABLE 1:** Anthropometric and metabolic parameters of the study population

	Lower PCSK9	Higher PCSK9	*p* value
Women:men	7: 6	8: 6	
Plasma PCSK9 (ng/ml)	**199.0 ± 13.5**	**317.1 ± 27.1**	**0.001**
Anthropometric parameters			
Age (years)	57.6 ± 1.8	58.4 ± 1.9	0.761
BMI (kg/m^2^)	34.1 ± 1.8	30.6 ± 0.9	0.089
Total fat mass (kg)	39.1 ± 4.2	33.1 ± 2.2	0.211
Android fat mass (kg)	3.98 ± 0.53	3.32 ± 0.36	0.303
Gynoid fat mass (kg)	5.90 ± 0.57	5.35 ± 0.40	0.432
Android‐to‐gynoid fat ratio	0.67 ± 0.06	0.64 ± 0.06	0.658
% Body Fat (%)	40.2 ± 2.1	40.2 ± 1.8	0.996
Waist circumference (cm)	110.2 ± 6.0	104.0 ± 3.3	0.362
Hip circumference (cm)	112.1 ± 6.2	109.6 ± 1.6	0.684
Energy intake and expenditure			
Basal metabolic rate (kcal/day)	1568 ± 105	1433 ± 87	0.331
Energy intake (kcal/day)^a^	2074 ± 205	2047 ± 122	0.907
% Fat^a^	33.4 ± 2.0	34.4 ± 1.0	0.642
% Carbohydrate^a^	48.8 ± 2.2	50.2 ± 1.4	0.571
% Protein^a^	16.4 ± 1.0	15.1 ± 0.7	0.254
% Saturated fat^a^	10.2 ± 0.7	10.7 ± 0.7	0.681
% Alcohol^a^	2.33 ± 0.87	1.09 ± 0.44	0.204
Sugar (g)^a^	86.7 ± 8.8	89.5 ± 8.5	0.826
Fiber (g)^a^	22.6 ± 2.2	27.1 ± 2.4	0.188
Fasting metabolic parameters			
Systolic blood pressure (mm Hg)	128.1 ± 3.6	118.9 ± 5.9	0.196
Diastolic blood pressure (mm Hg)	79.5 ± 2.4	74.5 ± 3.4	0.234
Plasma total cholesterol (mmol/L)	4.60 ± 0.13	4.78 ± 0.17	0.432
Plasma non‐HDL‐C (mmol/L)	3.47 ± 0.15	3.09 ± 0.13	0.066
Plasma LDL‐C (mmol/L)	2.66 ± 0.15	2.66 ± 0.13	1.000
Plasma HDL‐ C (mmol/L)	**1.13 ± 0.07**	**1.68 ± 0.17**	**0.007**
Plasma TGs (mmol/L)	**1.79 ± 0.25**	**0.96 ± 0.08**	**0.003**
Plasma NEFAs (mmol/L)	0.57 ± 0.05	0.48 ± 0.04	0.185
Plasma apoB (g/L)	**0.97 ± 0.06**	**0.81 ± 0.05**	**0.039**
Plasma apoB‐to‐PCSK9 (mg/µg)	**5.24 ± 0.53**	**2.72 ± 0.23**	**<0.001**
Plasma LDLC‐to‐PCSK9 (µmol/µg)	**14.0 ± 1.1**	**8.9 ± 0.6**	**<0.001**
11 in the higher PCSK9g/L)	1.41 ± 0.04	1.63 ± 0.10	0.067
Plasma glucose (mmol/L)	**4.77 ± 0.11**	**5.21 ± 0.13**	**0.017**
Plasma insulin (µU/ml)	18.2 ± 3.6	15.1 ± 2.5	0.478
Plasma C‐peptide (ng/ml)	1.97 ± 0.28	1.83 ± 0.26	0.701
HOMA‐IR (mmol/L × mU/L)	3.95 ± 0.80	3.48 ± 0.56	0.633

Data are presented as mean ± SEM.

^a^ For N = 11 in the lower PCSK9 group for uncompleted food journals. Significant data hold when performing sensitivity non‐parametric analysis. N.B. All metabolic parameters were measured on the 1st testing day prior to the Botnia clamp.

## 3 RESULTS

Baseline characteristics of the study groups with lower and higher plasma PCSK9 are presented in Table 1. Subjects with lower plasma PCSK9 had higher fasting plasma TGs, apoB, apoB‐to‐PCSK9 ratio, and LDLC‐to‐PCSK9 ratio and lower plasma HDL‐C and glucose. Notably, average plasma glucose and HDL‐C are within the normal ranges, and average plasma apoB is less than the 50^th^ percentile of the Canadian population (Connelly et al., 1999). There were no group differences in adiposity, body composition (% fat) or fat distribution (android and gynoid fat, android‐to‐gynoid fat ratio, waist, and hip circumferences), basal metabolic rate, energy intake, % macronutrient intake, % alcohol intake, or sugar and fiber intake. There was also no group‐difference in total fasting plasma NEFAs, cholesterol, non‐HDL‐C, LDL‐C or apoA‐1.

### 3.1 Subjects with lower plasma PCSK9 have higher WAT surface‐expression of LDLR and CD36 and activation of WAT NLRP3 inflammasome

White adipose tissue plasma membrane localization of LDLR and CD36 (termed WAT‐surface expression) was measured as previously published (Cyr et al., 2020). Compared to subjects with higher plasma PCSK9, subjects with lower plasma PCSK9 had significantly higher WAT‐surface expression of LDLR (+81%, Figure 2a) and CD36 (+36%, Figure 2b). There was no significant group‐difference in WAT NLRP3 protein expression (Figure 2c). However, subjects with lower plasma PCSK9 had 66% lower WAT pro‐IL‐1β protein expression than subjects with higher PCSK9 (Figure 2d), 284% higher WAT secretion of active IL‐1β *ex vivo* over 4 h (Figure 2e), and 85% higher plasma IL‐1Ra (Figure 2f). This suggests higher cleavage of pro‐IL‐1β into its active IL‐1β form and systemic activation of the IL‐1 pathway in subjects with lower plasma PCSK9. Adjustment for WAT surface‐expression of LDLR or CD36 alone did not eliminate group differences in WAT NLRP3 inflammasome priming (pro‐IL‐1β protein) or activation (WAT IL‐1β secretion) nor in plasma IL‐1Ra (Table 2). Similarly, adjusting for LDLR and CD36 ligands, plasma apoB or NEFAs, did not eliminate group differences in these inflammatory parameters. Concomitant adjustment for two or more of these covariates is not possible considering the sample size.

**TABLE 2:** Univariate ANOVA comparing subjects with lower versus higher plasma PCSK9 with adjustment for WAT‐surface expression of LDLR or CD36, plasma apoB or plasma NEFAs

	WAT‐surface expression of LDLR (% of CTL)	WAT‐surface expression of CD36 (% of CTL)	Plasma apoB (g/L)	Plasma NEFAs (mmol/L)
	*p*‐value			
WAT Pro‐IL−1β protein (% of CTL)^a^	**0.030**	**0.026**	**0.031**	**0.031**
WAT IL−1β secretion (pg/mg WAT)^b^	**0.015**	**0.027**	**0.015**	**0.026**
Plasma IL−1Ra (ng/ml)^c^	**0.044**	**0.042**	**0.021**	**0.040**
Log_10_ [WAT function]^c^	0.103	0.068	0.073	0.081
1^st^ phase DI (1st phase C‐peptide × M/I) ((ng/mL)*(mg/kg/min)/(uU/mL)	0.087	**0.039**	**0.047**	0.081
Total DI (Total C‐peptide × M/I) ((ng/mL)*(mg/kg/min)/(uU/mL)	0.076	0.077	**0.018**	0.072
Postprandial plasma TG curves (mmol/L)	—			
Group effect	0.285	0.080	0.114	0.063
Group × time interaction	0.184	**0.021**	**0.021**	**0.010**
Plasma TGs at 6 hours	—	**0.034**	**<0.001**	**0.026**

*p* value in bold represents significant values.

^a^ N = 12 in the higher PCSK9 group.

^b^ N = 12 in the lower and N = 9 in the higher PCSK9 group.

^c^ N = 12 in the higher PCSK9 group due to missing samples.

### 3.2 Subjects with lower plasma PCSK9 have lower WAT function, delayed postprandial plasma clearance of fat, and lower disposition index

As hypothesized, compared to subjects with higher plasma PCSK9, subjects with lower plasma PCSK9 and higher WAT surface‐expression of LDLR and CD36 had lower WAT function, assessed as lower in situ LPL activity in fresh WAT over 4 hours *ex vivo* (−62%, Figure 3a). As WAT function data were skewed, we performed a Log_10_ transformation and verified the group‐difference in WAT function, which was significant (*p* = 0.031). Moreover, subjects with lower PCSK9 had delayed postprandial plasma clearance of TGs over 6 hours after the ingestion of the high‐fat meal (by 2‐way RM ANOVA; group *p* = 0.040, Figure 3b). There was also a group × time interaction and a group‐difference in plasma TG at 6 h (*p* = 0.003). There were no group‐differences in 1st phase or total glucose‐induced insulin secretion (*p* > 0.05), 1st phase or total C‐peptide secretion (Figure 3c) or insulin sensitivity assessed as GIR (data not shown) or M/I during the Botnia clamp (Figure 3d). However, subjects with lower plasma PCSK9 had 37% lower 1st phase DI and 28% lower total DI than subjects with higher PCSK9 (Figure 3e‐f). This suggests that insulin secretion is less sufficient to compensate for insulin resistance in this group.

Adjustment for WAT‐surface expression of LDLR eliminated all group‐differences in risk factors for T2D (Log_10_ WAT function, 1st and total DI and postprandial hypertriglyceridemia), while adjustment for WAT‐surface expression of CD36 eliminated group‐differences in all risk factors except 1st phase DI and plasma TGs at 6 h (Table 2). Moreover, adjustment for plasma apoB as a measure of the number of apoB‐lipoproteins, mostly as LDL, eliminated group‐differences in Log_10_ WAT function and postprandial plasma clearance of TGs, while adjustment for plasma NEFAs eliminated all differences except in plasma TGs at 6 h (Table 2).

### 3.3 Effect of native human LDL on SGBS adipocyte differentiation, function, and inflammation

Whether human adipocytes express *NLRP3* mRNA is controversial (Koenen et al., 2011; Stienstra et al., 2010; Vandanmagsar et al., 2011). To explore the mechanism by which apoB‐lipoproteins may regulate human WAT function and inflammation, we compared the differentiation, function, and inflammation of human SGBS preadipocytes at baseline and after 7 days (7d) of differentiation in the absence or presence of low to physiological concentrations of human native LDL. Of note, the preadipocyte yield from human WAT biopsies is too small to complete multiple cycles of proliferation without losing differentiation capacity, hence the use of SGBS cells.

As presented in Figure 4, 7d‐differentiation of SGBS preadipocytes is associated with about a 40% decrease in cell number (Figure 4a,c) and a 770% increase in lipid‐droplet accumulation per cell (Figure 4b,c). Moreover, chronic exposure to LDL during differentiation increased cell number by about 100% (Figure 4d) and decreased lipid‐droplet accumulation per cell by about 50% in a dose‐dependent manner, reaching a plateau at 0.2–0.4 g/L of apoB (Figure 4e). Moreover, chronic exposure to native LDL inhibited adipocyte function measured as reduced in situ LPL activity, reaching a plateau of inhibition by 60% at 0.2 g/L of apoB (Figure 4f). Notably, 0.2–0.4 g/L of apoB represents lower than the 5th percentile of plasma apoB concentrations in the Canadian population (Connelly et al., 1999).

We further measured the expression of 20 key genes related to differentiation, function, and the NLRP3 inflammasome in the absence or presence of LDL (0.05 and 0.20 g apoB/L). Normal differentiation of SGBS preadipocytes upregulated the mRNA expression of peroxisome‐proliferator‐activated receptor γ (*PPARG)* and CCAT/enhancer‐binding protein‐α (*CEBPA*), but downregulated *CEBPD* (Figure 5A). It also upregulated the expression of key genes related to lipid metabolism (lipoprotein lipase [*LPL*], diacylglycerol acyltransferase [*DGAT*], adipose tissue triglyceride lipase [*ATGL*], acetyl‐CoA carboxylase 1 [*ACC1*], sterol regulatory element‐binding factors 1 and 2 [*SREBF1*, *SREBF2*], *LDLR* and *CD36*, Figure 5b) and to glucose metabolism and insulin action (adiponectin, glucose transporter 4, and hexokinase 2 [*ADIPOQ*, *GLUT4*, and *HK2*], Figure 5c). There was a detectable expression of *PCSK9* in preadipocytes, which is under the regulation of *SREBP2* (Dubuc et al., 2004) that was further increased upon differentiation (Figure 5b). However, we could not detect PCSK9 measured by immunoblot in the medium (data not shown). *NLRP3* expression was not detected in SGBS preadipocytes or adipocytes, with or without a 4‐hour incubation with LPS (0.1–1 ng/ml). Differentiation of SGBS cells markedly suppressed the expression of monocyte chemoattractant protein‐1 (*MCP*‐*1*), caused a minor but significant reduction in *IL1B* expression, but increased caspase‐1 (*CASP1*) expression, which is required for normal adipocyte differentiation (Stienstra et al., 2010) (Figure 5d).

Differentiation of SGBS was consistently affected in the presence of LDL in the differentiation medium generally in a dose‐dependent manner. Native LDL inhibited the expression of differentiation gene *CEBPA* (Figure 5a). They also inhibited the expression of genes related to lipid metabolism (*LPL*, *DGAT*, *ATGL*, *SREBF1*, *SREBF2*, *PCSK9*, *LDLR*, and *CD36*, Figure 5b) and to glucose metabolism and insulin action (*ADIPOQ* and *HK2*, Figure 5b,c). Finally, native LDL suppressed the expression of *CASP1* and markedly upregulated *MCP*‐*1*, particularly at 0.20 g apoB/L (Figure 5d).

## 4 DISCUSSION

In this analysis examining overweight and obese subjects with normal plasma LDL‐C separated based on median plasma PCSK9 per sex, we report that subjects with lower plasma PCSK9 identifying subjects with higher WAT‐surface LDLR and CD36 have (a) activation of WAT NLRP3 inflammasome measured as higher secretion of IL‐1β, higher plasma IL‐1Ra and lower WAT expression of pro‐IL‐1β; (b) higher risk factors for T2D assessed as lower WAT function, delayed postprandial plasma clearance of TGs and lower DI. Adjustment for WAT‐surface expression of LDLR or CD36 separately eliminated group‐differences in risk factors for T2D more than adjusting for their ligands (plasma apoB and NEFAs). However, adjustment for each receptor separately did not eliminate group‐differences related to WAT or systemic NLRP3 inflammasome activation. Low and physiological concentrations of native human LDL impair human SGBS adipocyte differentiation and function in a dose‐dependent manner, an effect that was independent of adipocyte NLRP3 inflammasome.

It should be underscored prior to discussion that this is a post hoc analysis of pooled data from two separate studies. A clinical trial was recently initiated in our lab that was designed to test the novel findings between subjects with lower and higher WAT surface‐expression of LDLR and CD36 identified using plasma PCSK9 that was unveiled by this analysis (clinical trial registry NCT04485871). Moreover, strength of this work is complementing observational data in human WAT with cellular studies in human adipocytes using native LDL. However, while native LDL was isolated from healthy young volunteers and kept in EDTA until used, it cannot be excluded that LDL preparations may have contained oxLDL or other toll‐like receptor ligands like LPS, which may have potentiated or increased LDL effects on adipocyte differentiation, function, and inflammation.

Data analysis emerging since 2010 reported that statin therapy is associated with ~12% higher incidence of diabetes in a dose‐dependent manner (Corrao et al., 2014; Ridker et al., 2012; Sattar et al., 2010; Swerdlow et al., 2015). Statins act by inducing the expression of LDLR (Preiss & Sattar, 2015). Further evidence linking the upregulated LDLR pathway to T2D emerged in 2015 using genetic data. One Mendelian‐randomization study in ~113,000 subjects with average plasma LDL‐C of 3.36 mmol reported that LDL‐lowering variants in *HMGCR*, *PCSK9*, and *LDLR* were associated with a 10–13% rise in diabetes risk for every 0.26 mM decrease in plasma LDL‐C (Ference et al., 2016). Another study in 568,448 subjects (average plasma LDL‐C of 3.41 mmol/L) reported an increase of 0.09 mmol/L in fasting plasma glucose and 29% in the diabetes risk for every 1 mmol/L decrease in plasma LDL‐C attributed to 4 *PCSK9* LOF variants (Schmidt et al., 2016). Taking into account the allele frequency of these variants and their T2D odds ratio, it can be deducted that the majority of T2D patients carry at least one allele of these variants. For example, ~77% of patients with T2D carry at least one copy of *PCSK9* allele rs2479409 (Schmidt et al., 2016) and ~84% of T2D patients are carriers of at least one copy of *HMGCR* allele rs12916 (Lotta et al., 2016). Notably, the addition of anti‐PCSK9 to statins in the FOURIER trial (using Evolocumab over a median of 2.2 years) (Sabatine et al., 2017) or in the ODYSSEY trial (using Alirocumab over a median of 2.8 years) (Ray et al., 2019) did not increase in the risk for T2D compared to statins alone. However, evidence from longer trials targeting PCSK9 are still awaited (Mascitelli & Goldstein, 2018; Schmidt et al., 2016).

Subjects with normal plasma LDL‐C (<3.5 mmol/L) were examined in this work to mimic plasma LDL‐C concentrations found in human conditions with LDL‐C lowering variants such as *PCSK9* loss‐of‐function variants and to focus inter‐subject variability on WAT expression of LDLR and CD36. Our findings indicate that subjects with higher WAT‐surface expression of LDLR and CD36, identified by lower plasma PCSK9, have an accumulation of WAT and systemic activation of NLRP3 inflammasome and related risk factors for T2D compared to subjects with higher PCSK9 and lower WAT LDLR and CD36 despite lack of group differences in body composition, basal metabolic rate, and energy and macronutrient intake.

WAT NLRP3 inflammasome is believed to link obesity to T2D (Koenen et al., 2011; Skeldon et al., 2014; Stienstra et al., 2010; Vandanmagsar et al., 2011). Stienstra *et al* first reported in 2010 that adipogenesis and insulin sensitivity were increased in human preadipocytes treated with IL‐1Ra, and IL‐1β antibodies, and in preadipocytes from *Nlrp3*−/− or *Casp1*−/− mice (Stienstra et al., 2010). Moreover, ablation of *Nlrp3* protected mice against high fat‐diet induced IR in WAT and liver (Vandanmagsar et al., 2011). In humans, targeting IL‐1β by recombinant IL‐1Ra (Larsen et al., 2007) or anti‐IL‐1β (Cavelti‐Weder et al., 2012; Sloan‐Lancaster et al., 2013) improves plasma glucose, *β*‐cell function, insulin resistance, and/or inflammation in T2D patients. As subjects with upregulated WAT‐surface expression of LDLR and CD36 have upregulated WAT NLRP3 inflammasome, our findings may explain why human conditions with inherited or induced upregulation of LDLR pathway are linked to higher risk of diabetes. Notably, adjusting for WAT‐surface expression of LDLR, as opposed to CD36 or plasma apoB and NEFAs, had the greatest effect on eliminating group‐differences in WAT function, postprandial plasma clearance of TG and DI. Intriguingly, despite the reported roles of CD36 (Sheedy et al., 2013), LDLR (Rampanelli et al., 2017), native LDL (Bissonnette, Lamantia, et al., 2018; Rampanelli et al., 2017), and NEFAs (Wen et al., 2011) in the upregulation of the NLRP3 inflammasome in cellular models and human WAT (Bissonnette, Lamantia, et al., 2018), adjustment for any of these parameters separately did not eliminate group difference in WAT NLRP3 inflammasome priming or activation nor in plasma IL‐1Ra. This may be related to the additive effect and interaction of the two receptors with their ligands to upregulate WAT NLRP3 inflammasome in subjects with lower plasma PCSK9 that could not be mimicked using concomitant adjustment for all variables in this analysis given the small sample size.

Controversy exists regarding the cell population within WAT that is responsible for NLRP3 inflammasome activity, being attributed to the stromal vascular fraction alone (Vandanmagsar et al., 2011), or to the adipocytes as well (Koenen et al., 2011). Thus, we examined the expression of NLRP3 in SGBS cells. We could not detect *NLRP3* mRNA in SGBS adipocytes, and to our knowledge, *NLRP3* mRNA or protein expression has not yet been reported in these cells. This suggests that the source of increased WAT IL‐1β secretion in subjects with lower plasma PCSK9 and upregulate WAT LDLR and CD36 pathways, and after the incubation of human WAT with native LDL (Bissonnette, Lamantia, et al., 2018), may not be adipocytes but resident macrophage within WAT. In line with this hypothesis, we recently reported macrophage infiltration of WAT in the fasting and 4‐hour postprandial states in a similar population of subjects with overweight and obesity (Cyr et al., 2020). Lower fasting plasma PCSK9 also predicted the increase in WAT macrophage infiltration in the postprandial state in this population (Cyr et al., 2020). The interaction between native LDL with LDLR, CD36, and PCSK9 in the regulation of NLRP3 inflammasome in primary human macrophages and adipocytes is currently being investigated.

Nevertheless, independent of the NLRP3 inflammasome, chronic exposure of SGBS cells to native LDL reduced their differentiation. This effect is likely to occur at a later stage of differentiation as the expression of *CEBPB* and *CEBPD*, which are expressed early in the differentiation process upregulating the expression of *PPARG* (Hamm et al., 2001), was unaffected by LDL treatment. Moreover, LDL downregulated *CEBPA*, which is expressed later in the differentiation process and is required, together with PPARγ, for the transcriptional activation of adipogenic genes, as shown in murine 3T3‐L1 adipocytes (Lefterova et al., 2008). Moreover, very low concentrations of LDL were sufficient to inhibit the expression of various genes involved in adipocyte function, lipid and glucose metabolism and insulin action. These low concentrations of LDL applied to SGBS cells also upregulated inflammation (*MCP*‐*1)*, which would explain lower adipocyte lipid accumulation and function in LDL‐treated preadipocytes. This may be a reflection of reduced differentiation, a consequence of excess LDL uptake, or both. Given that about 10% of adipocytes are believed to die annually and be replaced by an equal number of differentiating preadipocytes (Arner et al., 2010), and that impaired adipocyte differentiation plays a major role in WAT dysfunction, lipotoxicity, and cardiometabolic risk (Medina‐Gomez et al., 2007; Sauer, 2015), increased LDL uptake into preadipocytes of subjects with upregulated WAT‐surface expression of LDLR and CD36 pathway may promote reduced WAT function. LDL‐induced increases in *MCP*‐*1* expression in SGBS cells may also favor macrophage infiltration of WAT in these subjects, perpetuating the activation of NLRP3 inflammasome and reduced adipocyte differentiation during WAT turnover.

In conclusion, normocholesterolemic subjects with lower plasma PCSK9 and higher WAT‐surface expression of LDLR and CD36 have higher NLRP3 inflammasome activation and secretion of IL‐1β, systemic activation of IL‐1 system, lower WAT function, delayed postprandial plasma clearance of TGs and lower DI. This may explain, at least in part, the association of human conditions with naturally upregulated LDLR pathway with increased risk for T2D. The effect of LDL uptake on human WAT may be mediated by LDL‐induced reduction in adipocyte differentiation and function and increased *MCP*‐*1* expression. This we propose would fuel the cross‐talk between adipocytes and macrophages promoting WAT inflammation and dysfunction.

## CONFLICT OF INTEREST

The authors declare no conflict of interest associated with this manuscript.

## AUTHOR CONTRIBUTION

MF and YC designed research; YC, VL, SB, MC, MF conducted research. YC, SB, VL, MB, ABP, AD, MW, GM, JLE, MS, MF analyzed the data; YC and MF wrote the manuscript; all authors reviewed and contributed to the manuscript. MF is the guarantor of this work.

## References

[phy214721-bib-0001] Arner, E. , Westermark, P. O. , Spalding, K. L. , Britton, T. , Ryden, M. , Frisen, J. , Bernard, S. , & Arner, P. (2010). Adipocyte turnover: Relevance to human adipose tissue morphology. Diabetes, 59, 105–109. 19846802 10.2337/db09-0942 PMC2797910

[phy214721-bib-0002] Bissonnette, S. , Lamantia, V. , Cyr, Y. , Provost, V. , Devaux, M. , & Faraj, M. (2018). Native LDL are priming signals for the NLRP3 inflammasome in human white adipose tissue. International Symposium on Atherosclerosis.Atherosclerosis Supplements.

[phy214721-bib-0003] Bissonnette, S. , Saint‐Pierre, N. , Lamantia, V. , Cyr, Y. , Wassef, H. , & Faraj, M. (2015). Plasma IL‐1Ra: linking hyperapoB to risk factors for type 2 diabetes independent of obesity in humans. Nutr Diabetes, 5, e180. 26417659 10.1038/nutd.2015.30 PMC4657760

[phy214721-bib-0004] Bissonnette, S. , Saint‐Pierre, N. , Lamantia, V. , Leroux, C. , Provost, V. , Cyr, Y. , Rabasa‐Lhoret, R. , & Faraj, M. (2018). High plasma apolipoprotein B identifies obese subjects who best ameliorate white adipose tissue dysfunction and glucose‐induced hyperinsulinemia after a hypocaloric diet. The American Journal of Clinical Nutrition, 108, 62–76. 29917037 10.1093/ajcn/nqy070

[phy214721-bib-0005] Bissonnette, S. , Salem, H. , Wassef, H. , Saint‐Pierre, N. , Tardif, A. , Baass, A. , Dufour, R. , & Faraj, M. (2013). Low density lipoprotein delays clearance of triglyceride‐rich lipoprotein by human subcutaneous adipose tissue. Journal of Lipid Research, 54, 1466–1476. 23417739 10.1194/jlr.P023176 PMC3622338

[phy214721-bib-0006] Calvo, D. , Gómez‐Coronado, D. , Suárez, Y. , Lasunción, M. A. , & Vega, M. A. (1998). Human CD36 is a high affinity receptor for the native lipoproteins HDL, LDL, and VLDL. Journal of Lipid Research, 39, 777–788. 9555943

[phy214721-bib-0007] Cavelti‐Weder, C. , Babians‐Brunner, A. , Keller, C. , Stahel, M. A. , Kurz‐Levin, M. , Zayed, H. , Solinger, A. M. , Mandrup‐Poulsen, T. , Dinarello, C. A. , & Donath, M. Y. (2012). Effects of gevokizumab on glycemia and inflammatory markers in type 2 diabetes. Diabetes Care, 35, 1654–1662. 22699287 10.2337/dc11-2219 PMC3402269

[phy214721-bib-0008] Connelly, P. W. , Poapst, M. , Davignon, J. , Lussier‐Cacan, S. , Reeder, B. , Lessard, R. , Hegele, R. A. , & Csima, A. (1999). Reference values of plasma apolipoproteins A‐I and B, and association with nonlipid risk factors in the populations of two Canadian provinces: Quebec and Saskatchewan. Canadian Heart Health Surveys Research Group. Canadian Journal of Cardiology, 15, 409–418. 10322250

[phy214721-bib-0009] Corrao, G. , Ibrahim, B. , Nicotra, F. , Soranna, D. , Merlino, L. , Catapano, A. L. , Tragni, E. , Casula, M. , Grassi, G. , & Mancia, G. (2014). Statins and the risk of diabetes: Evidence from a large population‐based cohort study. Diabetes Care, 37, 2225–2232. 24969582 10.2337/dc13-2215

[phy214721-bib-0010] Cyr, Y. , Bissonnette, S. , Lamantia, V. , Wassef, H. , Loizon, E. , NGO Sock, E. T. , Vidal, H. , Mayer, G. , Chretien, M. , & Faraj, M. (2020). White adipose tissue surface expression of LDLR and CD36 is associated with risk factors for type 2 diabetes in adults with obesity. Obesity (Silver Spring), 28, 2357–2367. 33043593 10.1002/oby.22985

[phy214721-bib-0011] Cyr, Y. , Wassef, H. , Bissonnette, S. , Lamantia, V. , Davignon, J. , & Faraj, M. (2016). White adipose tissue apoC‐I secretion; role in delayed chylomicron clearance in vivo and ex vivo in white adipose tissue in obese subjects. Journal of Lipid Research, 57, 1074–1085. 27040450 10.1194/jlr.P064170 PMC4878191

[phy214721-bib-0012] Demers, A. , Samami, S. , Lauzier, B. , Des rosiers, C. , Sock, E. T. N. , Ong, H. , & Mayer, G. (2015). PCSK9 induces CD36 degradation and affects long‐chain fatty acid uptake and triglyceride metabolism in adipocytes and in mouse liver. Arteriosclerosis, Thrombosis, and Vascular Biology, 35, 2517–2525. 10.1161/ATVBAHA.115.306032 26494228

[phy214721-bib-0013] Dubuc, G. , Chamberland, A. , Wassef, H. , Davignon, J. , Seidah, N. G. , Bernier, L. , & Prat, A. (2004). Statins upregulate PCSK9, the gene encoding the proprotein convertase neural apoptosis‐regulated convertase‐1 implicated in familial hypercholesterolemia. Arteriosclerosis, Thrombosis, and Vascular Biology, 24, 1454–1459. 10.1161/01.ATV.0000134621.14315.43 15178557

[phy214721-bib-0014] Faraj, M. (2020). LDL, LDL receptors, and PCSK9 as modulators of the risk for type 2 diabetes: a focus on white adipose tissue. J Biomed Res, 34, 251–259. 32701068 10.7555/JBR.34.20190124 PMC7386410

[phy214721-bib-0015] Faraj, M. , Sniderman, A. , & Cianflone, K. (2004). ASP enhances i*n situ* lipoprotein lipase activity by increasing fatty acid trapping in adipocytes. Journal of Lipid Research, 45, 657–666. 14703506 10.1194/jlr.M300299-JLR200

[phy214721-bib-0016] Ference, B. A. , Robinson, J. G. , Brook, R. D. , Catapano, A. L. , Chapman, M. J. , Neff, D. R. , Voros, S. , Giugliano, R. P. , Davey Smith, G. , Fazio, S. , & Sabatine, M. S. (2016). Variation in PCSK9 and HMGCR and risk of cardiovascular disease and diabetes. New England Journal of Medicine, 375, 2144–2153. 10.1056/NEJMoa1604304 27959767

[phy214721-bib-0017] Fischer‐Posovszky, P. , Newell, F. S. , Wabitsch, M. , & Tornqvist, H. E. (2008). Human SGBS cells ‐ a unique tool for studies of human fat cell biology. Obesity Facts, 1, 184–189. 20054179 10.1159/000145784 PMC6452113

[phy214721-bib-0018] Gastaldelli, A. , Gaggini, M. , & Defronzo, R. A. (2017). Role of adipose tissue insulin resistance in the natural history of T2DM: Results from the San Antonio metabolism study. Diabetes, 66(4), 815–822. 28052966 10.2337/db16-1167

[phy214721-bib-0019] Glatz, J. F. C. , & Luiken, J. J. F. P. (2018). Dynamic role of the transmembrane glycoprotein CD36 (SR‐B2) in cellular fatty acid uptake and utilization. Journal of Lipid Research, 59(7), 1084–1093. 10.1194/jlr.R082933 29627764 PMC6027920

[phy214721-bib-0020] Hamm, J. K. , Park, B. H. , & Farmer, S. R. (2001). A role for C/EBPbeta in regulating peroxisome proliferator‐activated receptor gamma activity during adipogenesis in 3T3‐L1 preadipocytes. Journal of Biological Chemistry, 276, 18464–18471. 10.1074/jbc.M100797200 11279134

[phy214721-bib-0021] Koenen, T. B. , Stienstra, R. , van Tits, L. J. , Joosten, L. A. B. , van Velzen, J. F. , Hijmans, A. , Pol, J. A. , van der Vliet, J. A. , Netea, M. G. , Tack, C. J. , Stalenhoef, A. F. H. , & de Graaf, J. (2011). The inflammasome and caspase‐1 activation: A new mechanism underlying increased inflammatory activity in human visceral adipose tissue. Endocrinology, 152(10), 3769–3778. 21862623 10.1210/en.2010-1480

[phy214721-bib-0022] Lamantia, V. , Bissonnette, S. , Provost, V. , Devaux, M. , Cyr, Y. , Daneault, C. , Rosiers, C. D. , & Faraj, M. (2019). The association of polyunsaturated fatty acid delta‐5‐desaturase activity with risk factors for type 2 Diabetes Is Dependent On Plasma ApoB‐lipoproteins in overweight and obese adults. Journal of Nutrition, 149, 57–67. 10.1093/jn/nxy238 PMC6351138 30535058

[phy214721-bib-0023] Lamantia, V. , Bissonnette, S. , Wassef, H. , Cyr, Y. , Baass, A. , Dufour, R. , Rabasa‐Lhoret, R. , & Faraj, M. (2017). ApoB‐lipoproteins and dysfunctional white adipose tissue; Relation to risk factors for type 2 diabetes in humans. Journal of Clinical Lipidology, 11, 34–45. 28391908 10.1016/j.jacl.2016.09.013

[phy214721-bib-0024] Larsen, C. M. , Faulenbach, M. , Vaag, A. , Volund, A. , Ehses, J. A. , Seifert, B. , Mandrup‐Poulsen, T. , & Donath, M. Y. (2007). Interleukin‐1B receptor antagonist in type 2 diabetes mellitus. New England Journal of Medicine, 356, 1517–1526. 10.1056/NEJMoa065213 17429083

[phy214721-bib-0025] Lefterova, M. I. , Zhang, Y. , Steger, D. J. , Schupp, M. , Schug, J. , Cristancho, A. , Feng, D. , Zhuo, D. , Stoeckert Jr, C. J. , Liu, X. S. , & Lazar, M. A. (2008). PPARgamma and C/EBP factors orchestrate adipocyte biology via adjacent binding on a genome‐wide scale. Genes & Development, 22, 2941–2952. 18981473 10.1101/gad.1709008 PMC2577797

[phy214721-bib-0026] Lorenzo, C. , Wagenknecht, L. E. , Rewers, M. J. , Karter, A. J. , Bergman, R. N. , Hanley, A. J. G. , & Haffner, S. M. (2010). Disposition index, glucose effectiveness, and conversion to type 2 diabetes: The Insulin Resistance Atherosclerosis Study (IRAS). Diabetes Care, 33, 2098–2103. 20805282 10.2337/dc10-0165 PMC2928371

[phy214721-bib-0027] Lotta, L. A. , Sharp, S. J. , Burgess, S. , Perry, J. R. B. , Stewart, I. D. , Willems, S. M. , Luan, J. A. , Ardanaz, E. , Arriola, L. , Balkau, B. , Boeing, H. , Deloukas, P. , Forouhi, N. G. , Franks, P. W. , Grioni, S. , Kaaks, R. , Key, T. J. , Navarro, C. , Nilsson, P. M. , … Wareham, N. J. (2016). Association between LDL‐cholesterol lowering genetic variants and risk of type 2 diabetes. JAMA, 316, 1383–1391. 27701660 10.1001/jama.2016.14568 PMC5386134

[phy214721-bib-0028] Mascitelli, L. , & Goldstein, M. R. (2018). Questioning the safety and benefits of evolocumab. The Lancet Diabetes & Endocrinology, 6, 11. 10.1016/S2213-8587(17)30397-2 29273162

[phy214721-bib-0029] Medina‐Gomez, G. , Gray, S. , & Vidal‐Puig, A. (2007). Adipogenesis and lipotoxicity: role of peroxisome proliferator‐activated receptor gamma (PPARgamma) and PPARgammacoactivator‐1 (PGC1). Public Health Nutrition, 10, 1132–1137. 17903321 10.1017/S1368980007000614

[phy214721-bib-0030] Murphy, R. M. , & Lamb, G. D. (2013). Important considerations for protein analyses using antibody based techniques: down‐sizing Western blotting up‐sizes outcomes. Journal of Physiology, 591, 5823–5831. 10.1113/jphysiol.2013.263251 PMC3872754 24127618

[phy214721-bib-0031] Preiss, D. , & Sattar, N. (2015). Does the LDL receptor play a role in the risk of developing type 2 diabetes? JAMA, 313, 1016–1017. 25756436 10.1001/jama.2015.1275

[phy214721-bib-0032] Rampanelli, E. , Orso, E. , Ochodnicky, P. , Liebisch, G. , Bakker, P. J. , Claessen, N. , Butter, L. M. , van den Bergh Weerman, M. A. , Florquin, S. , Schmitz, G. , & Leemans, J. C. (2017). Metabolic injury‐induced NLRP3 inflammasome activation dampens phospholipid degradation. Scientific Reports, 7, 2861. 28588189 10.1038/s41598-017-01994-9 PMC5460122

[phy214721-bib-0033] Ray, K. K. , Colhoun, H. M. , Szarek, M. , Baccara‐Dinet, M. , Bhatt, D. L. , Bittner, V. A. , Budaj, A. J. , Diaz, R. , Goodman, S. G. , Hanotin, C. , Harrington, R. A. , Jukema, J. W. , Loizeau, V. , Lopes, R. D. , Moryusef, A. , Murin, J. , Pordy, R. , Ristic, A. D. , Roe, M. T. , … INVESTIGATORS . (2019). Effects of alirocumab on cardiovascular and metabolic outcomes after acute coronary syndrome in patients with or without diabetes: a prespecified analysis of the ODYSSEY OUTCOMES randomised controlled trial. Lancet Diabetes Endocrinol, 7, 618–628. 31272931 10.1016/S2213-8587(19)30158-5

[phy214721-bib-0034] Ridker, P. M. , Pradhan, A. , Macfadyen, J. , Libby, P. , & Glynn, R. J. (2012). Cardiovascular benefits and diabetes risks of statin therapy in primary prevention: An analysis from the JUPITER trial. The Lancet, 380, 565–571. 10.1016/S0140-6736(12)61190-8 PMC3774022 22883507

[phy214721-bib-0035] Sabatine, M. S. , Leiter, L. A. , Wiviott, S. D. , Giugliano, R. P. , Deedwania, P. , de Ferrari, G. M. , Murphy, S. A. , Kuder, J. F. , Gouni‐Berthold, I. , Lewis, B. S. , Handelsman, Y. , Pineda, A. L. , Honarpour, N. , Keech, A. C. , Sever, P. S. , & Pedersen, T. R. (2017). Cardiovascular safety and efficacy of the PCSK9 inhibitor evolocumab in patients with and without diabetes and the effect of evolocumab on glycaemia and risk of new‐onset diabetes: a prespecified analysis of the FOURIER randomised controlled trial. The Lancet Diabetes & Endocrinology, 5, 941–950. 28927706 10.1016/S2213-8587(17)30313-3

[phy214721-bib-0036] Sattar, N. , Preiss, D. , Murray, H. M. , Welsh, P. , Buckley, B. M. , de Craen, A. J. M. , Seshasai, S. R. K. , McMurray, J. J. , Freeman, D. J. , Jukema, J. W. , Macfarlane, P. W. , Packard, C. J. , Stott, D. J. , Westendorp, R. G. , Shepherd, J. , Davis, B. R. , Pressel, S. L. , Marchioli, R. , Marfisi, R. M. , … Ford, I. (2010). Statins and risk of incident diabetes: a collaborative meta‐analysis of randomised statin trials. The Lancet, 375, 735–742. 10.1016/S0140-6736(09)61965-6 20167359

[phy214721-bib-0037] Sauer, S. , (2015). Ligands for the nuclear peroxisome proliferator‐activated receptor gamma. Trends in Pharmacological Sciences, 36, 688–704. 26435213 10.1016/j.tips.2015.06.010

[phy214721-bib-0038] Schmidt, A. F. , Swerdlow, D. I. , Holmes, M. V. , Patel, R. S. , Fairhurst‐Hunter, Z. , Lyall, D. M. , Hartwig, F. P. , Horta, B. L. , Hyppönen, E. , Power, C. , Moldovan, M. , van Iperen, E. , Hovingh, G. K. , Demuth, I. , Norman, K. , Steinhagen‐Thiessen, E. , Demuth, J. , Bertram, L. , Liu, T. , … Völker, U. et al (2016). PCSK9 genetic variants and risk of type 2 diabetes: A mendelian randomisation study. The Lancet Diabetes & Endocrinology, 5, 97–105. 27908689 10.1016/S2213-8587(16)30396-5 PMC5266795

[phy214721-bib-0039] Schmidt, R. J. , Beyer, T. P. , Bensch, W. R. , Qian, Y. W. , Lin, A. , Kowala, M. , Alborn, W. E. , Konrad, R. J. , & Cao, G. (2008). Secreted proprotein convertase subtilisin/kexin type 9 reduces both hepatic and extrahepatic low‐density lipoprotein receptors in vivo. Biochemical and Biophysical Research Communications, 370, 634–640. 18406350 10.1016/j.bbrc.2008.04.004

[phy214721-bib-0040] Sheedy, F. J. , Grebe, A. , Rayner, K. J. , Kalantari, P. , Ramkhelawon, B. , Carpenter, S. B. , Becker, C. E. , Ediriweera, H. N. , Mullick, A. E. , Golenbock, D. T. , Stuart, L. M. , Latz, E. , Fitzgerald, K. A. , & Moore, K. J. (2013). CD36 coordinates NLRP3 inflammasome activation by facilitating intracellular nucleation of soluble ligands into particulate ligands in sterile inflammation. Nature Immunology, 14, 812–820. 23812099 10.1038/ni.2639 PMC3720827

[phy214721-bib-0041] Skeldon, A. M. , Faraj, M. , & Saleh, M. (2014). Caspases and inflammasomes in metabolic inflammation. Immunology and Cell Biology, 92, 304–313. 24518981 10.1038/icb.2014.5

[phy214721-bib-0042] Sloan‐Lancaster, J. , Abu‐Raddad, E. , Polzer, J. , Miller, J. W. , Scherer, J. C. , de Gaetano, A. , Berg, J. K. , & Landschulz, W. H. (2013). Double‐blind, randomized study evaluating the glycemic and anti‐inflammatory effects of subcutaneous LY2189102, a neutralizing IL‐1β antibody, in patients with type 2 diabetes. Diabetes Care, 36, 2239–2246. 23514733 10.2337/dc12-1835 PMC3714510

[phy214721-bib-0043] Stienstra, R. , Joosten, L. A. B. , Koenen, T. , van Tits, B. , van Diepen, J. A. , van den Berg, S. A. A. , Rensen, P. C. N. , Voshol, P. J. , Fantuzzi, G. , Hijmans, A. , Kersten, S. , Müller, M. , van den Berg, W. B. , van Rooijen, N. , Wabitsch, M. , Kullberg, B. J. , van der Meer, J. W. M. , Kanneganti, T. , Tack, C. J. , & Netea, M. G. (2010). The inflammasome‐mediated caspase‐1 activation controls adipocyte differentiation and insulin sensitivity. Cell Metabolism, 12, 593–605. 21109192 10.1016/j.cmet.2010.11.011 PMC3683568

[phy214721-bib-0044] Swanson, K. V. , Deng, M. , & Ting, J. P. (2019). The NLRP3 inflammasome: molecular activation and regulation to therapeutics. Nature Reviews Immunology, 19, 477–489. 10.1038/s41577-019-0165-0 PMC7807242 31036962

[phy214721-bib-0045] Swerdlow, D. I. , Preiss, D. , Kuchenbaecker, K. B. , Holmes, M. V. , Engmann, J. E. L. , Shah, T. , Sofat, R. , Stender, S. , Johnson, P. C. D. , Scott, R. A. , Leusink, M. , Verweij, N. , Sharp, S. J. , Guo, Y. , Giambartolomei, C. , Chung, C. , Peasey, A. , Amuzu, A. , Li, K. , …, Delaney, J. A. et al (2015). HMG‐coenzyme A reductase inhibition, type 2 diabetes, and bodyweight: evidence from genetic analysis and randomised trials. The Lancet, 385, 351–361. 10.1016/S0140-6736(14)61183-1 PMC4322187 25262344

[phy214721-bib-0046] Vandanmagsar, B. , Youm, Y. H. , Ravussin, A. , Galgani, J. E. , Stadler, K. , Mynatt, R. L. , Ravussin, E. , Stephens, J. M. , & Dixit, V. D. (2011). The NLRP3 inflammasome instigates obesity‐induced inflammation and insulin resistance. Nature Medicine, 17, 179–188. 10.1038/nm.2279 PMC3076025 21217695

[phy214721-bib-0047] Wassef, H. , Bissonnette, S. , Saint‐Pierre, N. , Lamantia, V. , Cyr, Y. , Chretien, M. , & Faraj, M. (2015). The apoB/ PCSK9 ratio: a new index for metabolic risk in humans. Journal of Clinical Lipidology, 9, 664–675. 26350813 10.1016/j.jacl.2015.06.012

[phy214721-bib-0048] Wassef, H. , Salem, H. , Bissonnette, S. , Baass, A. , Dufour, R. , Davignon, J. , & Faraj, M. (2012). White adipose tissue‐apoC‐I secretion; relation to delayed plasma clearance of dietary fat in humans. Arterioscler Throm Vasc Biol, 32, 2785–2793. 10.1161/ATVBAHA.112.300306 22995522

[phy214721-bib-0049] Wen, H. , Gris, D. , Lei, Y. , Jha, S. , Zhang, L. , Huang, M.‐T.‐H. , Brickey, W. J. , & Ting, J.‐P.‐Y. (2011). Fatty acid‐induced NLRP3‐ASC inflammasome activation interferes with insulin signaling. Nature Immunology, 12, 408–415. 21478880 10.1038/ni.2022 PMC4090391

